# RNA proximity sequencing data and analysis pipeline from a human neuroblastoma nuclear transcriptome

**DOI:** 10.1038/s41597-020-0372-3

**Published:** 2020-01-28

**Authors:** Steven W. Wingett, Simon Andrews, Peter Fraser, Jörg Morf

**Affiliations:** 1 Laboratory of Nuclear Dynamics, Babraham Institute, Cambridge, UK; 2 Bioinformatics, Babraham Institute, Cambridge, UK; 3 Department of Biological Science, Florida State University, Tallahassee, FL USA

**Keywords:** Transcriptomics, Data processing, RNA sequencing

## Abstract

We have previously developed and described a method for measuring RNA co-locations within cells, called Proximity RNA-seq, which promises insights into RNA expression, processing, storage and translation. Here, we describe transcriptome-wide proximity RNA-seq datasets obtained from human neuroblastoma SH-SY5Y cell nuclei. To aid future users of this method, we also describe and release our analysis pipeline, CloseCall, which maps cDNA to a custom transcript annotation and allocates cDNA-linked barcodes to barcode groups. CloseCall then performs Monte Carlo simulations on the data to identify pairs of transcripts, which are co-barcoded more frequently than expected by chance. Furthermore, derived co-barcoding frequencies for individual transcripts, dubbed valency, serve as proxies for RNA density or connectivity for that given transcript. We outline how this pipeline was applied to these sequencing datasets and openly share the processed data outputs and access to a virtual machine that runs CloseCall. The resulting data specify the spatial organization of RNAs and builds hypotheses for potential regulatory relationships between RNAs.

## Abstract (summary)

**Table:** 

Measurement(s)	RNA • Proximity
Technology Type(s)	RNA sequencing
Factor Type(s)	biological replicate
Sample Characteristic - Organism	Homo sapiens

Machine-accessible metadata file describing the reported data: 10.6084/m9.figshare.11627397

## Background & Summary

The positioning of RNA molecules and the spatial organization of transcriptomes in cells is poorly understood to date. Nascent RNA emerges from its encoding gene during synthesis, and genome folding and gene positioning therefore determines the spatial point of origin for any RNA molecule. After completion of transcription and co-transcriptional processing, a molecule can diffuse freely in nuclei^1^. Most protein-coding RNAs will be exported swiftly from nuclei. Some transcripts however reside within nuclei and are frequently localized in proximity of their encoding gene. This is achieved by chromatin retention through interactions between chromatin-bound proteins or the genome itself and the RNA molecule or by a very short half-life of the RNA and its prompt degradation in the close vicinity of the gene^2^. Such spatial restraints can have implications for RNAs regulating the expression of other genes, by limiting the number of accessible target genes in 3-dimensional space to the immediate neighbourhood of the regulatory RNA. For example, Xist RNA represses transcription specifically of one X chromosome thereby relying on the local retention to the X chromosome territory^3,4^. Malat1 and Neat1 are architectural, relatively stable, non-coding RNAs integral to membraneless and transcription-permissive bodies scattered throughout the nucleoplasm^5,6^. However, these structures are assembled co-transcriptionally at the gene loci of the respective non-coding RNAs^7,8^. The property of RNA and proteins to separate from surroundings and to form bodies to compartmentalize cellular tasks can further specify RNA localization but becomes detrimental for cells when mutations in molecules resident to such structures are acquired that lead to irreversible aggregation and disease states, as observed for example for RNA repeats^9^.

The elucidation of the spatial nuclear organization based on sequencing has been pushed forward by chromosome conformation capture HiC^10,11^. However, DNA-sparse nuclear regions frequently escape chromosome conformation measurements. For example, a major phase-separated compartment in the nucleus, the nucleolus, encompasses up to 10% of the nuclear volume but has been virtually invisible to HiC, due to the low overall genome but high repeat sequence content within these structures. To capture such DNA-sparse blind spots we devised Proximity RNA-seq, which identifies the co-localization of RNA pairs and groups through in-droplet barcoding of RNA molecules in subcellular, crosslinked particles. The method applied to nuclei identifies RNA-containing structures as exemplified by nucleoli and other bodies, estimates relative distances of transcripts to such cellular landmarks and provides a proxy of local RNA density as reported in the accompanying publication^12^.

Here we describe the structure and characteristics of Proximity RNA-seq datasets obtained from human cell nuclei and provide further information on the analysis. In particular, we explain the rationales behind the computational steps and describe output files at different stages and the usage of the pipeline. Three biological replicates were separately generated and analysed before being merged into a large dataset in order to derive statistical significance estimates for RNA proximities through the comparison with Monte Carlo randomisations. The datasets can be re-analysed by the reader, and aid in building hypotheses on spatial RNA organization and regulation within cells. For users to become familiar with CloseCall, we set up a publicly accessible virtual machine and provide a test dataset (https://osf.io/mwd73)^13^. The virtual machine, running a Linux operating system, has CloseCall and all its dependencies and genome files installed. Finally, we propose RNA network visualizations, which represent RNA-RNA proximities and RNA density.

## Methods

**Fig. 1:**
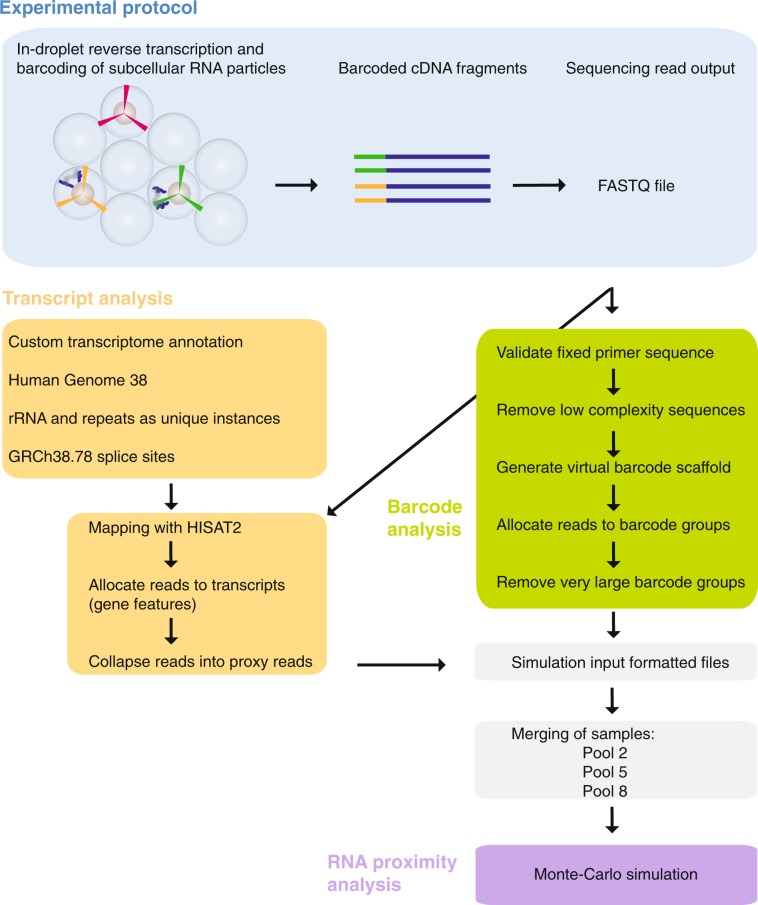
Workflow of Proximity RNA-seq, CloseCall and sample processing. Reverse transcription and RNA co-barcoding in droplets generate sequencing libraries with cDNAs, whose RNA templates were in spatial proximity, sharing the same barcode (blue box). After Illumina sequencing, single-end sequence reads from the FASTQ file are validated to contain a defined primer sequence. Barcodes of cDNAs are extracted and, if near-identical, grouped together (green box). In parallel, the cDNA part of each read is mapped onto the genome and allocated to a custom transcriptome annotation (yellow box). The Monte-Carlo simulation takes merged datasets as input and randomises cDNA – barcode pairings to derive expected co-barcoding frequencies for RNA pairs.

We present here in more detail the computational procedures to analyse Proximity RNA-seq data, which follows the description of all experimental steps as reported in the accompanying manuscript^12^ (Fig. 1). Furthermore, we outline how to generate RNA proximity networks that visualize proximity significance and RNA valency for transcripts, the latter being an estimate of local RNA density or connectivity.

### Experimental part of proximity RNA-seq

#### Sample preparation

Datasets presented here were obtained from human, neuron-like SH-SY5Y cells. Cultures were grown in high-glucose DMEM medium (Thermo Fisher Scientific) containing 10% fetal bovine serum (Hyclone) and Streptomycin, Penicillin (Thermo Fisher Scientific). Cells were crosslinked at 70–80% confluency. The culture medium of cells growing in a 15 cm dish was replaced by 19.8 ml pre-warmed 1x PBS, and 0.2 ml freshly prepared 10 mM ethylene glycolbis(succinimidylsuccinate) (EGS, Thermo Fisher Scientific) in DMSO was added drop-wise for 1 mM final concentration. Cells were incubated for 10 min at 37 °C. 1.4 ml 16% formaldehyde (Agar Scientific) were subsequently added to the 15 cm dish for a final concentration of 1% and the incubation was extended for another 10 min at room temperature. To quench crosslinking 3.5 ml 1 M glycine were added and cells were scraped immediately and pelleted in a 50 ml falcon tube at 210 × g for 5 min at 4 °C. Cell pellets were washed once with 1x PBS supplemented with 125 mM glycine, once with 1x PBS only. Cell pellets were flash frozen in liquid nitrogen and stored at −80 °C until use.

Cell pellets, processed as one pellet from a single 15 cm dish per tube, were thawed on ice in 1 ml 20 mM Tris pH 7.2, spun and resuspended in ice-cold 1.5 ml hypotonic lysis buffer (20 mM Tris, pH 7.2, 5 mM NaCl, 0.2% Igepal C-630, 2 mM EDTA, 1 mM EGTA) supplemented with 1x Complete, EDTA-free protease inhibitor cocktail (Roche Applied Science) and 0.5 units/µl SUPERase IN RNase inhibitor (Thermo Fisher Scientific). Cells resuspended in lysis buffer were incubated for 30 min on ice with occasional mixing. The homogenate was spun at 2 krpm for 5 min in a benchtop centrifuge at room temperature to enrich nuclei. Pelleted nuclei were then resuspended in SDS washing buffer (20 mM Tris, pH 7.2, 5 mM NaCl, 0.3% SDS, 2 mM EDTA, 1 mM EGTA) supplemented with inhibitors as listed for the lysis buffer and incubated for 10 min at room temperature in a thermoblock with constant mixing at 750 rpm. Then Triton X-100 was added for a final concentration of 1.7% and the incubation continued for 10 min. Nuclei were washed once in 10 mM Tris pH 7.2, 5 mM NaCl, 0.5 mM EDTA, 1% Triton X-100, supplemented with inhibitors as specified above, and once in the same buffer with only 0.05% Triton X-100. Nuclei were resuspend in 0.2 ml wash buffer with 0.05% Triton X-100 and sonicated in 15 ml falcon tubes using a bioruptor UCD-200 sonicator (Diagenode) with power set to medium and cycles of 10 seconds “on” followed by 10 seconds “off” at 8 °C. The integrity of nuclei was inspected after each sonication cycle by light microscopy and using trypan blue staining. Sonication was stopped once the large majority of nuclei were disrupted, usually after 3–5 cycles. Sonicated nuclear homogenates were flash frozen in liquid nitrogen and stored at −80 °C until use.

To estimate RNA and DNA content and fragment length crosslinks in samples were reversed and nucleic acids purified. To extract DNA, 20 µl of nuclear homogenate were supplemented with the following reagents to final concentrations of 50 mM Tris pH 8.0, 50 mM NaCl, 2 mM EDTA, 0.2% SDS and with 5 µl proteinase K (Roche Applied Science, 10 mg/ml stock concentration) for a total volume of 40 µl. DNA extraction mixes were first incubated for 4 hours at room temperature followed by 2 hours at 70 °C. 2 µl RNase A (Roche Applied Science, 10 mg/ml stock concentration) was added and samples were incubated for 1 hour at 37 °C. DNA was then purified by phenol/chloroform extraction, followed by salt/isopropanol precipitation and resuspended in 10 µl nuclease-free water (Ambion). To extract RNA, the following reagents were added to 20 µl of nuclear homogenate to reach final concentrations of 100 mM sodium citrate pH 6.2, 2 mM EDTA, 0.2% SDS and supplemented with 5 µl proteinase K for a total volume of 40 µl. RNA extraction mixes were incubated for 4 hours at room temperature followed by 1 hour at 70 °C. 1 ml Trizol LS (Thermo Fisher Scientific) was added and RNA isolated according to the manufacturer’s manual and resuspended in 10 µl water. RNA and DNA content and fragment length were measured by a spectrophotometer (NanoDrop) and agarose gel electrophoresis.

#### Preparation of barcoded, random primer-containing beads

High-complexity barcode libraries on magnetic beads were generated by PCR amplification in emulsion droplets of individual random DNA barcode templates on beads with one of the two PCR primers immobilized.

To generate two batches of barcoded beads, approximately 1.2 × 10^8^ magnetic streptavidin beads (17 µl of MyOne C1 beads, Thermo Fisher Scientific, at 7–10 × 10^6^/µl) were transferred into a polypropylene tube (Treff), captured on a magnetic rack and washed in high salt buffer (20 mM Tris pH 8.0, 1 M NaCl, 1 mM EDTA). The beads were then resuspended in 35 µl high salt buffer and 6 µl dual-biotin PCR primer R (100 µM stock, Table 1) were added and the mixture was vortexed. After binding for 20 min at room temperature, beads were washed twice in high salt buffer and once in TTLEB buffer (10 mM Tris pH 8.0, 0.5 mM EDTA, 0.05% Triton X-100, and 0.04 µg/µl molecular biology grade BSA, NEB). Beads were resuspended in 35 µl TTLEB buffer and stored at 4 °C.

**Table 1:** Primers and adapters used in Proximity RNA-seq.

	Modification	Sequence (5′-3′)	Purification
**Bead barcoding**			
Primer-F		CCATCTCATCCCTGCGTGTC	HPLC
Primer-R		CCTATCCCCTGTGTGCCTTG	HPLC
Primer-R	5′ dual biotin	CCACTACGCTCGCTATCCTATCCCCTGTGTGCCTTG	HPLC
Random barcode template		CCATCTCATCCCTGCGTGTCNNNNNNNNNNNNNNNNNNNNNNNNNNGATCGTCGGACTGTAGAACTCCCTATAGTGAGTCGTATTACAAGGCACACAGGGGATAGG	PAGE
Random tail primer F		NNNNNNNNNNNNNNNCCATCTCATCCCTGCGTGTC	HPLC
**Bead quality control (FACS)**			
T7 probe	5′ Cy5	CGTCGGACTGTAGAACTCCCTATAGTGAGTCGTA	HPLC
**Library preparation/amplification**			
PolyC12 primer		GCCTTGGCACCCGAGAATTCCACCCCCCCCCCCC	PAGE
RP1		AATGATACGGCGACCACCGAGATCTACACGTTCAGAGTTCTACAGTCCGA	PAGE
Index 6		CAAGCAGAAGACGGCATACGAGATATTGGCGTGACTGGAGTTCCTTGGCACCCGAGAATTCCA	PAGE
Index 12		CAAGCAGAAGACGGCATACGAGATTACAAGGTGACTGGAGTTCCTTGGCACCCGAGAATTCCA	PAGE
Index 5		CAAGCAGAAGACGGCATACGAGATCACTGTGTGACTGGAGTTCCTTGGCACCCGAGAATTCCA	PAGE
Index 19		CAAGCAGAAGACGGCATACGAGATTTTCACGTGACTGGAGTTCCTTGGCACCCGAGAATTCCA	PAGE

The PCR mix for one batch of beads (approximately 0.6 × 10^8^ beads) was prepared on ice in a 1.5 ml DNA LoBind tube (Eppendorf) by combining 943 µl water, 128 µl 10x PCR buffer I (AccuPrime, Thermo Fisher Scientific), 25 µl dNTPs (stock: 25 mM dNTP each), 32 µl MgSO4 (stock: 50 mM MgSO4, from AccuPrime kit), 32 µl primer F (100 µM stock), 40 µl of 1 μM non-biotinylated primer R (Table 1), 35 µl beads covered with immobilized dual-biotin PCR primer R and 20 µl AccuPrime Taq polymerase. The mix was pipetted extensively. 25 µl 1 nM random barcode template was heated for 1 min at 95 °C and directly added to the PCR mix on ice. The mix was again extensively pipetted on ice. 8 polypropylene tubes (Treff) with each containing 480 µl Pico-Surf™ 1–5% in Novec 7500 (Sphere Fluidics) were prepared. To 480 µl Pico-Surf 160 µl of the PCR mix was added per tube. The mixtures were then emulsified by vortexing using a vortex genie 2 vortexer (Scientific Industries) on a horizontal tube holder at 4 °C for 20 min at maximum speed. 50 µl aliquots of the emulsion were pipetted into wells of a 96-well hard-shell plate (Biorad). The PCR program used included 1 min at 94 °C, 35 cycles with 15 sec at 94 °C, 30 sec at 58 °C, 45 sec at 68 °C, followed by 5 min at 68 °C. The PCR reactions were pooled into eight 1.5 ml tubes and spun at 2 krpm for 1–2 min in a benchtop centrifuge or until the non-emulsified oil phase appeared at the bottom. The lower oil phase was removed and 480 µl 5% Ficoll 400 was added to each tube and the tubes vortexed. Then 350 µl PFOH (1H,1H,2H,2H-Perfluorooctan-1-ol) was added to each tube and the mixture was briefly vortexed and incubated at 37 °C for 5 min. To separate beads from the mixture, the tubes were placed in a magnetic rack, which was then incubated at 37 °C for 5–10 min. The PFOH phase at the bottom and the aqueous upper phase, if transparent, were removed. Beads were resuspended in the remaining aqueous phase and TLE buffer (10 mM Tris pH 8.0, 0.5 mM EDTA) was added for a total volume of around 300 µl. The PFOH extraction and magnetic separation were repeated. Beads were then washed 3–4x in TLE buffer and transferred into new tubes. The following washes were then carried out: 3x in 1% SDS buffer (20 mM Tris pH 8.0, 5 mM NaCl, 1% SDS), 1x in TLE buffer supplemented with 1% Triton X-100 (TTLE), 1x in TTLEB buffer. The beads from different tubes were pooled throughout the washes and resuspended in 50 µl TTLEB buffer.

According to a Poisson distribution, if around 50% of the total of beads are covered with copies of barcodes, approximately 70% of the 50% barcoded beads have copies of a single barcode and 30% have copies of multiple barcodes. To control whether a batch of beads contains a fraction of around 50% of barcoded beads, suitable to perform Proximity RNA-seq experiments, bead barcoding was measured by flow cytometry, FACS, analysis. 2 µl of a bead batch resuspended in 50 µl TTLEB were added to 3 µl 10x Accuprime PCR buffer I, 23 µl water and 2 µl of T7 Cy5-fluorescent probe (100 uM) and the sample was mixed by pipetting. The sample was incubated in a thermocycler for 2 min at 94 °C, 2 min at 80 °C, followed by incubations for 2 min at every degree from 75 °C to 61 °C, 10 min at 60 °C and 2 min at 55 °C, 50 °C and 45 °C. Beads were washed once in high salt buffer, resuspended in 1x Accuprime PCR buffer I, pre-warmed at 67 °C, and incubated for 3 minutes at 67 °C. The washes and incubations were repeated two more times. The fraction of barcoded beads was then determined by FACS with settings for APC 670/14 nm. Batches with 40–60% barcoded beads passed quality control.

Random tails were then added to 3′ ends of barcodes in bulk reactions for each bead batch. First, unused primers on beads were digested by an exonuclease I treatment (NEB, 650 µl total reaction including 65 µl exonuclease I) at 37 °C for 90 min. Beads were then washed 1x in 1% SDS buffer, 1x in TLE buffer with 1% Triton X-100 and 1x in TTLEB. To remove untemplated adenosine overhangs added by Taq polymerase, beads were incubated for 30 min at 20 °C in 10 µl NEBNext End Repair Reaction Buffer, 5 µl NEBNext End Repair Enzyme Mix (NEB) and 85 µl water. Wash steps were repeated as outlined after exonuclease I treatment. Beads were then subjected to a T7 exonuclease treatment in 20 µl NEB 4 buffer, 10 µl T7 exonuclease (NEB) and 170 µl water for 15 min at 25 °C to generate single-stranded barcodes immobilized via their dual-biotinylated 3′ ends, which are inert to exonuclease activity. The same wash steps as described above were repeated. To add random bases to barcode ends on beads, a primer with a 5′ random overhang was hybridized to the primer sequence at the 3′ end of barcodes, and the overhang used as template to introduce 3′ random bases to barcodes. Beads were resuspended in a Pfx polymerase mix (Thermo Fisher Scientific) consisting of 10 µl 10x Platinum Pfx buffer, 84 µl water, 3 µl 10 mM nucleotides, 2 µl 50 mM MgSO4, 1 µl random tail primer (100 µM, Tables 1) and 1 µl Pfx polymerase enzyme. The mix was incubated for 2 min at 94 °C, 5 min at 55 °C and 10 min at 68 °C. Beads were washed as described above after exonuclease I treatment. The T7 exonuclease treatment to generate single-stranded barcodes was repeated as described above. After washes, beads were resuspend in 50 µl TTLEB and stored at −20 °C until use.

#### In-emulsion reverse transcription of RNA-containing particles and crosslink reversal

Two batches of beads were used for one Proximity RNA-seq experiment with nuclear homogenate equivalent to 50 ng of reverse-crosslinked and purified RNA. The beads were resuspended in 30 µl TTLEB and freshly prepared Actinomycin D (Sigma) in DMSO was added for a final concentration of 6 ng/µl to inhibit reverse transcriptase activity on DNA templates. Possible aggregates of beads were disrupted by extensive pipetting, followed by sonication using a bioruptor UCD-200 sonicator (Diagenode) with power set to medium for 2 cycles of 5 sec “on” followed by 5 sec “off”. A 160 µl reverse transcription (RT) mix (Superscript III kit, Thermo Fisher Scientific) contained 32 µl 5X First-Strand Buffer, 8 µl 10 mM dNTP Mix (10 mM each nucleotide), 8 µl 0.1 M DTT, 2 µl RNase Inhibitor, 0.32 µl BSA for a final concentration of 0.04 µg/µl (NEB), 1.6 µl of MgCl_2_ for a final concentration of 0.5 mM, 16 µl Superscript III, 2 µl Actinomycin D with a final concentration of 5 ng/µl, nuclear homogenate corresponding to 50 ng RNA and water. The beads were heated at 90 °C for 1 min before the addition to the RT mix on ice. The emulsion was prepared with 550 µl Pico-Surf on a vortexer as described above at 4 °C for 40 min at maximum speed. 50 µl of emulsion was added per well to a 96-well plate. A cycling temperature program was used for reverse transcription. In a thermocycler with lid temperature at 55 °C, samples were incubated at 55 °C for 2 min, then placed on ice for 2 min followed by 30 min at 22 °C. Then the following cycle was repeated 60 times: 22 °C for 1 min, ramping to 50 °C with a rate of 0.5 °C/second, 50 °C for 1 min. Emulsions were then broken as described earlier. Beads were resuspended in 200 µl of 20 mM Tris pH 8.0, 50 mM NaCl and incubated overnight at 65 °C. cDNA on beads was treated with 1 µl RNase H (Thermo Fisher Scientific) and 0.5 µl RNase A (Roche Applied Science, 10 mg/ml stock concentration) in 5 µl 10x RNaseH buffer l (Thermo Fisher Scientific) and 43.5 µl water for 30 min at 37 °C. Beads were then washed as described above and resuspended in TTLEB.

#### Proximity RNA-seq library preparation

3′ cDNA ends on beads were dGTP/ddGTP-tailed to provide complementarity for the C-tailed, second Illumina primer sequence. The first Illumina primer site is present as a flanking sequence 5′ to the barcode in random barcode templates. Beads were resuspended in 5 µl 10x TdT buffer (NEB), 5 µl cobalt chloride, 2.5 µl of 100 µM dGTP/ddGTP mix (95 µM dGTP, Thermo Fisher Scientific, 5 µM ddGTP, Sigma)), 5 µl TdT enzyme (2 units/µl final) and 32.5 µl water and incubated for 20 min at 37 °C. Beads were washed 1 × 1% SDS buffer, 1x in TLE buffer with 1% Triton X-100, 1x in 10 mM Tris pH 8.0, 0.5 M NaCl, 0.5 mM EDTA, 0.05% Triton X-100, 2x in TTLEB. cDNA was then amplified using Accuprime HiFi Taq pol (Thermo Fisher Scientific). Beads were resuspended in 5 µl 10x Accuprime buffer I, 42.5 µl water, 0.5 µl RP1_long primer (50 µM stock, Table 1), 0.5 µl polyC12_cDNA adapter (50 µM stock), 0.5 µl Accuprime HiFi Taq pol (Thermo Fisher Scientific). After 1 min incubation at 94 °C, 5 cycles of 15 sec at 94 °C, 45 sec at 52 °C, 2 min 30 sec at 68 °C were carried out. The beads were captured, the supernatant containing the barcoded cDNA library transferred to a new tube and 150 µl of 20 mM Tris pH 8, 50 mM NaCl, 1 mM EDTA added. Size selection of PCR products using AMPure XP beads (Beckman-Coulter, 0.75x the sample volume added) was repeated twice. An emulsion PCR followed as a second amplification step. The eluate in water from the pre-amplification PCR was mixed with 20 µl 10x Accuprime buffer I, 2 µl RP1_long (50 µM), 2 µl Index primer (50 µM), 1 µl dNTPs (stock of 25 mM each dNTP, Thermo Fisher Scientific), 1 µl MgSO4 (50 mM stock), 4 µl Accuprime HiFi Taq pol and additional water for a final volume of 200 µl. The mix was emulsified for 20 min with 600 µl Pico-Surf as described above. The emulsion was transferred into wells of a 96-well plate and the following PCR program run: 94 °C for 1 min, 20–24 cycles of 15 sec at 94 °C, 30 sec at 52 °C, 2 min 30 sec at 68 °C. After pooling the PCR reactions the aqueous phase was recovered and DNA size-selected with once 0.65x Ampure beads and a second round with 0.8x Ampure beads. The final libraries were eluted in 35 µl TLE buffer. The concentration and fragment size distribution of libraries, ranging from 0.3 to 1.2 kb with a peak around 0.7 kb, were determined by Bioanalyzer profiles (Agilent Technologies) and Kapa Illumina SYBR green qPCR (Kapa Biosystems) according to manufacturer’s instructions.

#### CloseCall analysis pipeline

The individual scripts of the pipeline are listed in Table 2 and its execution is described in Usage Notes. The following sections describe the details of the different analysis steps.

**Table 2:** CloseCall scripts.

Analysis Area (Fig. 1)	Step	Script Name	Input	Main Output
Transcript Analysis	1	create_features.pl	List of gene annotations and list of repeat elements	Transcript annotation file
		*Creates custom transcript annotation*		
Barcode Analysis	2	check_sequence_present.pl	FASTQ file	FASTQ file
		*Filters for reads containing the expected primer sequence after the barcode and before cDNA*		
Barcode Analysis	3	problem_barcodes.pl	FASTQ file from step 2	FASTQ file
		*Filters out barcodes with nucleotide biases or adapter sequence content*		
Barcode Analysis	4	map_trimmed_barcodes.pl	FASTQ file from step 3	SAM file
		*Creates a virtual barcode genome and maps barcodes back to this genome*		
Barcode Analysis	5	group_trimmed_barcodes.pl	SAM file from step 4	Text file of relationships
		*Establishes barcode group relationships*		
Barcode Analysis	6	assign_read_to_barcode_group.pl	FASTQ file from step 3 and relationships file from step 5	FASTQ file
		*Assigns barcodes to barcode groups*		
Transcript Analysis	7	trim.pl	FASTQ file from step 6	FASTQ file
		*Extracts high-quality cDNA read sequencs (50 bases)*		
Transcript Analysis	8	CloseCall (pipeline master script)	FASTQ file from step 7	FASTQ screen summary files
		*FastQ Screen runs directly from pipeline master script*		
Transcript Analysis	9	mapper_hisat2.pl	FASTQ file from step 8, genome index files, splice sites	SAM file
		*Maps reads with HISAT2*		
Transcript Analysis	10	map_editor.pl	SAM file from step 9 and list of repeat elements	BAM file
		*Filters reads into uniquely, multi-mapped or mapped to pre-specified repeats*		
Transcript Analysis	11	create_data_file_include_noninteracting.pl	BAM file from step 10	Interactions text file
		*De-duplicates identical reads from within the same barcode group and then removes barocode groups comprising a higher than expected number of members. Produces a list of barcode group numerical ids and associated read midpoint coordinates*.		
Summary Results	12	CloseCall (pipeline master script)	Interactions file from step 11	Summary results files
		*Collection of R scripts produce summary metrics on the dataset*		
Transcript Analysis	13	identify_reads_by_regions.pl	Interactions file from step 11	Interactions text file
		*Filters for reads mapping to pre-defined transcripts*		
Transcript Analysis	14	remove_gene_duplicates.pl	Interactions file from step 12	Interactions text file
		*Removes identical transcripts from within the same barcode group (i.e. creating proxy reads)*		
Transcript Analysis	15	format_for_simulation.pl	Interactions file from step 14 and list of repeat elements	Interactions text file
		*Repeat features present in multiple locations in the genome are now treated as one single entity. The data is also parsed to produce an output file suitable for the Monte Carlo Simulation*.		
Transcript Analysis	16	createDitags_features.pl	Text file from step 15	Pairwise interactions file
		*Transforms barcode groups into transcript-transcript pairwise interactions*		
Summary Results	17	calc_frequeny_interactions.pl	Text file from step 15	Summary results files
		*Creates summary statistics*		
Transcript Analysis	18	reporter.pl	Summary files generated by the previous steps as the pipeline proceeds	Summary results files
		*Collates and produces more summary results*		
Additional Script	Valency	feature_valency_distribution.pl	Text file from step 15	Text file listing frequencies
		*Reports the frequency of each feature, segmented by valency/barcode group size*		
Monte Carlo Simulation	Sim1	anacondamontecarlo.jar	Text file from step 15	Text file of the simulation results, comparing the observed interaction frequencies with those simulated.
		*Performs the specified number of Monte Carlo simulations*		
Monte Carlo Simulation	Sim2	collate_monte_carlo_results.pl	Simulation results from step Monte Carlo Simulation 1	Text file of the simulation results, comparing the observed interaction frequencies with those simulated.
		*Collates the results of Monte Carlo simulations run as separate jobs*		
Monte Carlo Simulation	Sim3	multiple_testing_correction.pl	Simulation results from step Monte Carlo Simulation (from either step Sim1 or step Sim2)	Text file listing statistical significance for RNA pairs
		*Compares the real/random and random/random simulation results to identify statistically significant interactions*		

The CloseCall pipeline comprises a series of Perl cripts, and a master script (named CloseCall), which executes in turn each of the other scripts as data proceeds from step to step. The table lists name and main functions (in italics) of the scripts in the pipeline, starting from the input FASTQ files produced by sequencing, to the file taken as input by the Monte Carlos simulation, and finally to the list of statistically significant RNA-RNA proximities.

#### Identification of canonical RNA-seq reads

**Fig. 2:**
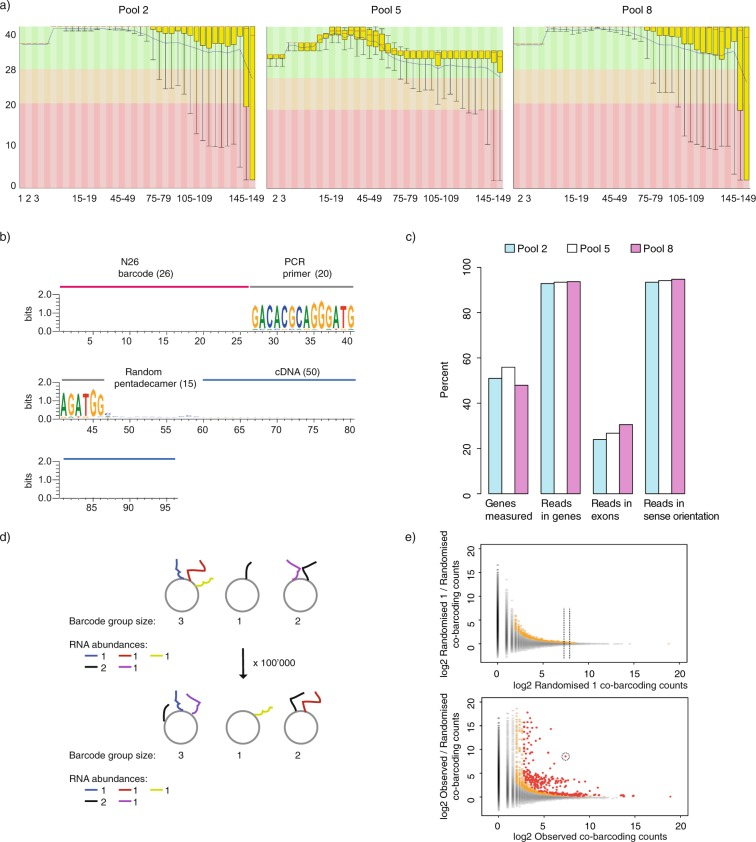
Quality of Proximity RNA-seq fragments, comparative read mapping statistics and randomisations. (**a**) Base calling quality scores (y axis) with background colours indicating very good (green), medium (orange) and poor (red) quality plotted against read position (x axis) using FASTQC (http://www.bioinformatics.babraham.ac.uk/projects/fastqc/). (**b)** Schematic of a sequenced Proximity RNA-seq library fragment with base distributions along raw fragment sequences. Of note, only 50 bases of the cDNA part were subsequently used to ensure the usage of high base quality scores. (**c**) Read mapping statistics for different datasets. (**d**) Randomisations of RNA proximities taking transcript-specific abundances and barcode group sizes on beads into account. (**e**) Plots of observed to randomized co-barcoding count ratios against observed co-barcoding counts. Each dot represents one RNA-RNA pairing. The top panel depicts the co-barcoding ratio of a randomised data set (randomised 1) to the average of all its randomisations, which provides a background distribution. The bottom panel shows the ratio of actual, observed co-barcoding to the average of all its randomisations. In both plots, pairwise RNA proximities with at least 2 observations were included and coloured in grey. Coloured in yellow are proximities with at least 3 observations and a local background-corrected p value <= 0.01, in red are proximities with at least 3 observations and a Benjamini-Hochberg corrected p value <= 0.05.

Proximity RNA-seq libraries were sequenced on two or three sequencing lanes in order to ensure sufficient depth. FASTQ files of 150 bp single-end reads from the same library were merged into one FASTQ file. After assessing sequencing quality (Fig. 2a), genuine Proximity RNA-seq reads were selected. Details of Proximity RNA-seq cDNA constructs are described in Fig. 2b. The sequenced parts of a fragment include a 26-base random barcode, which should be unique to a single bead. After the barcode is a PCR primer, required for amplification of the barcode on the bead. The base distribution of raw sequences of such fragments is depicted in Fig. 2b and shows the defined PCR primer sequence. The PCR primer was used to select canonical constructs. CloseCall allowed for one base insertion or one deletion or one substitution for every 10 bases of the PCR primer. At the 3′ end of the PCR primer sequence is a random pentadecamer, of unique sequence for each barcode copy on a bead, which hybridizes onto RNA molecules to initiate reverse transcription of the RNA template. The sequence following the random pentadecamer is the cDNA copy of an RNA molecule, which can be aligned to the appropriate reference genome to determine its origin.

#### Virtual scaffold strategy for the allocation of barcodes to barcode groups

Barcodes were first trimmed to 20 bases by removing 3 bases at either end as we observed that sequences could be offset by typically one base. Barcodes with low-complexity sequence content, i.e. the same nucleotide 13 or more times in a 20 bp sequence, were discarded. Removal of low-complexity barcodes led to a reduction of the population of barcodes encompassing a very large, likely artifactual, group of transcripts. CloseCall furthermore applied a maximum barcode group size threshold. This threshold is determined by modelling the barcode group sizes as a Poisson distribution (i.e. many barcodes are observed with small barcode group sizes, only few barcodes have very large barcode group sizes). Barcode groups of a size so large that they would have been expected to occur with a probability of less than 0.001 were removed from the dataset. In addition, the pipeline filtered out barcode sequences similar to adapter sequences. The extraction of the barcode sequence enables a transcript to be allocated to a particular nuclear RNA-containing particle. However, since sequencing errors in barcodes do occur, we expected occasional splitting of actual, proximal RNAs into separate barcode groups. To overcome this problem, a virtual barcode scaffold of all the sequenced barcodes was created. All unique, trimmed barcode sequences were concatenated into a single virtual barcode scaffold, with each individual barcode separated by a stretch of 24 undetermined nucleotides (i.e. Ns). CloseCall used the alignment tool Bowtie2^14^ to generate genome index files using the newly created virtual barcode FASTA file as a reference. Each trimmed barcode was then mapped back to the reference using Bowtie2 (in default mode for FASTA sequences and reporting all alignments (-a)), generating a SAM format file of positions of barcode alignments on the reference barcode scaffold. The cases with multi-mapping of barcodes to the virtual barcode scaffold identified barcodes that derived from the same initial barcode sequence but likely acquired sequence changes throughout the experimental protocol or during sequencing. The positions on the virtual barcode scaffold that shared multi-mapped barcodes were collapsed into one barcode group.

#### Mapping to a custom transcriptome annotation

CloseCall extracted 50 bases of the putative cDNA from each read after the barcode, PCR primer and random priming sequence and used HISAT2^15^ to map cDNA with default parameters and known splice-sites (GRCh38.78) to split reads across exon junctions. After mapping, reads were assigned to transcripts. The inherent ambiguous organization of transcriptomes required a custom curated transcriptome annotation and a series of priority rules for overlapping transcripts^12^. CloseCall takes as input an annotation feature file, a tab-delimited text file listing ENSEMBL Human Genome 38 (Ensembl 78) entries with chromosome, start position, end position, strand and feature name of all genes/transcripts. rRNA repeat sequences were masked (i.e. bases were replaced with Ns) in the reference genome FASTA file. Then, a 45S-pre-rRNA sequence was incorporated in our genome assembly as a separate chromosome. On mapping, rRNA reads now aligned uniquely to that chromosome. Other repeat classes were not N-masked in the input FASTA file, but rather the locations of these repeat elements were marked in the custom genome annotation file CloseCall takes as input. Reads multi-mapping to these regions were allowed by the pipeline. Specifically, these regions were: 5S, HY1, HY3, HY4, HY5, U1, U2, U3, U4, U5, U6, U7, U8, U13, U14, U17, BC200, tRNA (all tRNAs were collapsed into one feature), 7SLRNA and 7SK. The genomic coordinates of these elements were obtained from repeat masker, Human Genome 38, http://www.repeatmasker.org. Uniquely mapping reads and reads multi-mapping to any of the repeat classes included in the annotation are retained by CloseCall. Other multi-mapping reads are discarded. To enable consistent allocation of reads to genomic features, we developed a series of rules for read-to-transcript assignments. Firstly, repeat regions took priority, even if they overlapped with other features. Consequently, any read overlapping a gene and region defined as a repeat was assigned to the repeat. After defining repeat regions, the forward and reverse strands were processed independently. Thus, a feature allocation to the forward strand was not represented on the reverse strand at the same coordinates. Regions in which two or more features overlapped on the same strand were considered ambiguous and were left unclassified and removed from the annotation. However, features contained wholly within another feature were prioritised. In such cases, the internal feature is used to specify the region of overlap. For example, many small-nucleolar RNAs are contained within larger genes and form an abundant component of nuclear Proximity RNA-seq datasets. After annotation processing, CloseCall produces a tab-delimited text file of the genome annotation to be used subsequently by the pipeline. Using the mapped coordinates, reads were then allocated to their relevant feature. Each transcript is only represented once per subcellular particle and barcode group as it cannot be distinguished whether a single RNA molecule has been primed and reverse-transcribed multiple times at different positions along the molecule or whether multiple copies of the same transcript were present. Consequently, if multiple identically barcoded reads mapped to the same feature, this was recorded as one transcript observation called a proxy read. Using proxy reads for the comparison of the fractions of detected genes and of reads within genes, overlapping exons and in sense orientation to the annotation between all three experiments showed very similar results (Fig. 2c).

#### Statistical analysis of RNA proximities

CloseCall identifies transcripts, which are detected in proximity to each other more often than expected by chance, by comparison of observed data to sample-specific randomised data.

When generating data randomisations, the pipeline takes into account differences in RNA abundance and the number of co-barcoded transcripts per bead, i.e. barcode group size (Fig. 2d). Firstly, the pipeline records the number of proxy reads for each transcript in a dataset and the barcode group size of each bead. Secondly, CloseCall generates a randomised dataset that matches the observed barcode group size distribution and transcripts expression levels. Figure 2d outlines this randomisation process.

A technical problem when generating such random datasets is that a given transcript could be allocated to the same barcode group more than once. As mentioned above, during analysis of the actual observed data, multiple reads of the same transcripts with the same barcode were collapsed into one proxy read. Therefore, if the same feature was assigned twice to the same barcode group in a randomisation, the second instance of the feature was rejected and the step of random feature selection and assignment was repeated. Since abundant features would be disproportionally more likely to be rejected by this process, this would lead to abundant features being under-represented in a final random dataset. To prevent this, rejected features were added first to the next randomly selected barcode group. CloseCall performed 100,000 randomisations of the observed dataset.

The vast differences in expression levels between all RNAs of a transcriptome, together with the encapsulation of RNA particles according to Poisson distribution into droplets, results in highly expressed transcripts to be found frequently co-barcoded by chance in the same droplet. Real, significant proximity between two abundant transcripts is therefore often characterised by low enrichments of observed to randomised co-barcoding counts but very small p values due to the high co-barcoding counts, i.e. large sample size (Fig. 2e). On the other hand, lowly expressed transcripts (with a small sample size) can show large observed to randomised co-barcoding count ratios with fewer co-barcoding counts. Thus, CloseCall first generated a “background distribution”, which describes local distributions of expected co-barcoding for different ranges of co-barcoding counts. One randomisation of the observed dataset served as input for 100,000 further randomisations. Figure 2e (top panel) shows how one randomly generated dataset (random 1) compared against the average values of its 100,000 randomisations. In contrast, the observed dataset compared against the average values of its 100,000 randomisations (Fig. 2e, bottom panel) showed frequently outliers, with higher observed to randomised co-barcoding compared to the background distribution. Importantly, and as stated above, ratios of outlier RNA-RNA pairs (Fig. 2e, bottom, y axis) decreased with increasing co-barcoding counts (x axis). To formally classify an RNA-RNA pairing in observed data as significant, we computationally transposed each data point from the graph depicting observed against randomised data (Fig. 2e, bottom panel) onto the background distribution (graph in top panel). For example, the encircled point on the lower panel of Fig. 2e was transposed onto the background distribution to decide whether this RNA pair co-localised significantly. CloseCall would compare the point against its local distribution (a slice of the surrounding 500 data points) in the background model. CloseCall then addressed whether the point of interest was significantly above its local distribution (collection of points falling between the two dotted lines on the top panel). To do this, the magnitudes of co-ordinate displacements of the surrounding 500 data points were modelled as a normal distribution. The position of the interaction of interest, with respect to that normal distribution, specified its p value. The more the point deviated above the mean of the normal distribution, the less likely it was to occur by chance. The derived p values underwent Bonferroni-Hochberg multiple-testing correction.

CloseCall generates multiple output and summary files as data progresses through the pipeline. Users of CloseCall should particularly take note of the following files:

Simulation formatted file (*simulation_formatted.txt.gz)

This file lists all barcoding and co-barcoding events obtained after performing the necessary mapping and quality control steps. It reports the barcode ID and transcript name and feature coordinates and serves as the Monte Carlo simulation input.

Mapping and QC summary file (*anaconda_summary_results.txt)

This file collates the key metrics into one file to allow the user to assess easily the quality of a Proximity RNA-seq dataset. The file reports the pipeline steps, such as the percentage of reads containing the fixed primer sequence, the number of unique barcode groups, the mapping efficiency and the degree of PCR duplication as shown in Table 3.

**Table 3:** Samples and read processing statistics.

Row:	Pipeline step:		p2 library	p5 library	p8 library
2	Fixed_Seq_Check:	Sequencing reads	90967875	235347955	132988019
3		Matching Fixed_Seq	85407381	221586324	112960113
4		%Matching Fixed_Seq	93.9	94.2	84.9
5	Barcode filtering:	LC & Adapter Pass	85213377	221124118	112733569
6		%LC & Adapter Pass	99.8	99.8	99.8
7		Unique barcodes	8508280	33612246	22583834
8		Barcode groups	7161395	30135828	20674495
9		Reads (row 5) allocated to barcode groups (row 8)	84883356	220848562	112687674
10	Mapping:	Uniquely mapped (incl. rRNA annotation)	72444996	174241156	72111191
11		Multi-mapped on RNA repeats	546788	1873537	625221
12		Multi-mapped (excl. row 11)	6609030	14837602	5038160
13		Unmapped	5282542	29896267	34913102
14		%Mapped	85.99	79.74	64.55
15	Deduplication:	Reads for further use (row 10 + 11)	72991784	176114693	72736412
16		%PCR deduplication, barcode group size limit (of row 15)	9.92	9.44	9.6
17		%Within annotation (of row 16)	92.89	93.55	94.84
18		%Proxy reads (of row 17)	90.48	93.59	89.27
19		Proxy reads (of row 17)	5888752	13988521	5627689
21	Summary:	Proxy reads (row 19)	5888752	13988521	5627689
22		Barcode groups with proxy reads (row 19)	5047022	12993806	4768049
23		Reads in barcode group 1	4323281	12116171	4059143
24		Reads in barcode group 2	1243424	1550542	1163214
25		Reads in barcode group 3	263778	267969	319842
26		Reads in barcode group 4	49468	45988	72452
27		Reads in barcode group 5	8075	7065	11970
28		Reads in barcode group 6	726	786	1068
29		%Proxy reads co-barcoded	26.6	13.4	14.8

Breakdown of read numbers from replicate libraries, pool 2, pool 5 and pool 8, sequenced at different steps of CloseCall processing. Fixed_Seq: Primer sequences for on-bead barcode amplification. LC: low complexity barcode sequences. Of note, Proximity RNA-seq libraries are sequenced close to saturation (approximately 10% of unique reads remain after deduplication) in order to increase the number of detected co-barcoding events. Up to 25% of proxy reads are co-barcoded.

Monte Carlo Simulation file (*simulation_collated_data.*.qval.txt.gz)

This file lists all the pairwise RNA proximities in a dataset, the frequency with which they occurred in the observed dataset and in the Monte Carlo simulations.

Valency table (*valency_feature_distribution.txt.gz)

The file lists each transcript and the number of times it was observed in barcode groups of size 1, 2, etc. Transcript valency is derived as described in the next section.

#### Assignment of valency to transcripts

The so-called valency is an estimate for transcript-specific RNA density or connectivity. Valency 1 represents the instances a transcript has been detected singly on a bead, valency 2 represents co-barcoding between two different transcripts on a bead, valency 3 stands for a total of 3 transcripts with the same barcode on a bead and so on. The Perl script feature_valency_distribution.pl uses the Simulation Formatted file, [*.simulation_formatted.txt.gz], and outputs a list containing each feature and the number of times it was observed as valency 1, valency 2 etc. We then selected transcripts with the sum of valency 1, 2 and 3 greater than 10 proxy reads. For each transcript, counts in valency 1, 2 and 3 were divided by the sum of all three valencies of that given transcript. Subsequently, the transcriptome-wide distributions of the three valencies were separately transformed into z-scores. Transcripts were then assigned to high and low valency classes, respectively. High valency transcripts were defined based on z-scores of valency 1 < 0 and a mean of valency 2 and 3 z-scores > 0. Low valency transcripts had z-scores of valency 1 > 0 and a mean of valency 2 and 3 z-scores < 0. Only transcripts assigned to high and low valency classes in pool 2, pool 5 and pool 8 Proximity RNA-seq datasets were retained^12^. The valency data is available on GEO, accession: GSE129732^16^, file: GSE129732_local_RNA_connectivity.xlsx, and lists valency 1, 2 and 3 z-scores for pool 2, 5 and 8 for each feature.

#### Dataset correlations

**Fig. 3:**
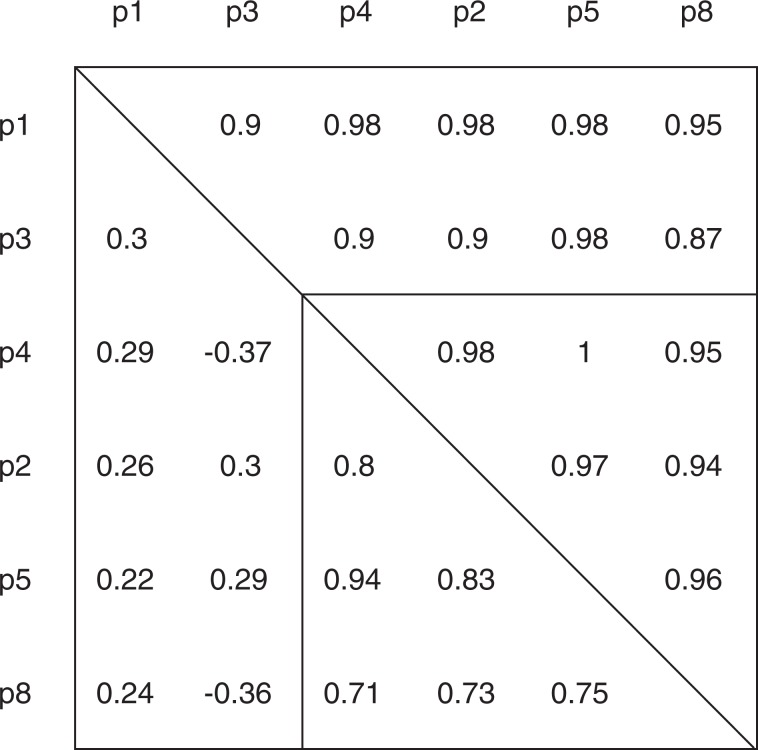
Pairwise Spearman rank correlation coefficients between different Proximity RNA-seq libraries. Correlations between different Proximity RNA-seq datasets using the number of proxy reads (upper, right triangle) or the number of detected co-barcoding events between two transcripts (lower, left triangle) as variables. Libraries p2, p5 and p8 are biological replicates with crosslinked nuclear homogenates from SH-SY5Y cells. p1 is a control library with randomly barcoded, non-clonal beads, p3 is a control with reverse-crosslinked RNA. p4 has been prepared in parallel and is comparable in sequencing depth to p1 and p3. Subsequently, p4 has been sequenced deeper to generate p5.

Correlation coefficients between different Proximity RNA-seq datasets using the number of proxy reads or the number of detected co-barcoding events between two transcripts as variables, shown in Fig. 3, were derived using pairwise Spearman rank correlations. To correlate datasets by transcript abundance we removed the top and bottom 10% transcripts based on expression, i.e. proxy read counts. For co-barcoding correlations between datasets, an initial cut-off of 5 or more observations for RNA pairs in all datasets was applied. Then datasets were trimmed by removing the top and bottom 10% pairs based on number of co-barcoding observations. Trimming removes highly abundant transcripts or RNA pairs, which inflate correlation coefficients, as well as very low transcript counts or spurious pairs, which lower coefficients.

#### Networks

**Fig. 4:**
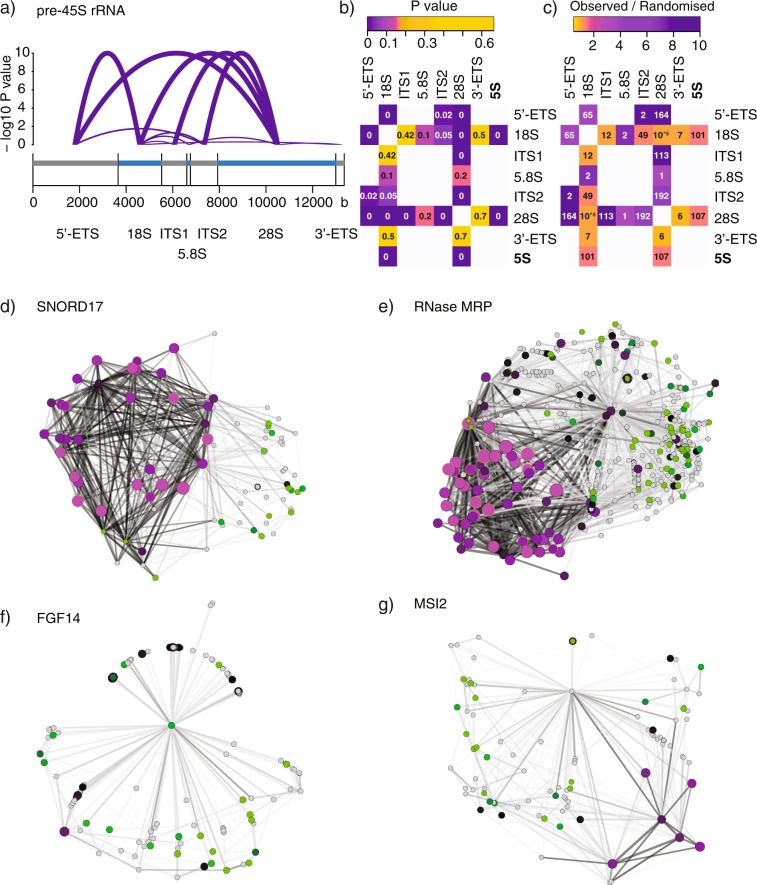
Validation of Proximity RNA-seq. (**a**) Transcript structure of pre-45S rRNA. RNA proximities between precursor parts and mature rRNA are indicated by arcs. Arc height and thickness represent – log10 p values with a plateau at 10. (**b**) Matrix of P values, indicated in matrix cells, for pre-45S rRNA and 5S proximities. White matrix cells signify pairs for which no data was obtained. (**c**) Matrix of observed/randomised co-barcoding count ratios. The colour gradient represents observed/randomised co-barcoding count ratios, the numbers in matrix cells are observed co-barcoding counts. (**d**–**g**) Candidate transcript-specific proximity networks (p value <= 0.1) in which thicker edge lines indicate proximities with lower p values, purple and bigger nodes represent higher valency transcripts, green nodes lower valency, black nodes middle valency. Grey nodes are transcript without assigned valency. (**d**) SNORD17 network. (**e**) RNase MRP. (**f)** FGF14. (**g**) MSI2.

Pairwise RNA proximities with p value < = 0.1 were imported into Cytoscape 3.7.1^17^ with valency assignments as a separate attribute file. Relative Entropy Optimization (EntOpt)^18^ on -log10 p values of RNA proximities was used for network layout. For each transcript the mean of valency 1, 2 and 3 was calculated for all three replicates (libraries p2, p5, p8). The average of the means then defined node size and colour. Node and edge size and colours are further specified in Fig. 4.

## Data Records

Sequenced data from Proximity RNA-seq experiments are available/stored on Gene Expression Omnibus accession: GSE129732^16^.

The GEO entry provides sequencing data and the following processed output files: GSE129732_Monte_Carlo_Simulation_Results.txt.gz lists all frequencies of observed and simulated RNA pairs and simulation-derived p values, GSE129732_Pools_2_5_8_simulation_format_combined_file.txt.gz reports all barcoding and co-barcoding events, listing barcode ID, transcript name and coordinates, and GSE129732_local_RNA_connectivity.xlsx lists transcript-specific valency, i.e. RNA density or connectivity.

Sequencing libraries p2, p5 and p8 are replicates from individual SH-SY5Y cell cultures. In Fig. 3 we present as well experimental control libraries. p1 is a control library for which we used randomly barcoded beads, i.e. beads with many different barcodes instead of copies of a single barcode sequence, p3 is a control library that has been performed with reverse-crosslinked RNA. p4 has been prepared in parallel to the controls p1 and p3. Subsequently, p4 has been sequenced deeper to generate p5. p2, p5 and p8 were pooled into one library for Monte Carlo simulations and further downstream analysis.

## Technical Validation

After data processing we first compared read quality and statistics of replicate datasets (pool 2, pool 5, pool 8, Fig. 2a). Good read quality scores were obtained for all samples and we found very similar fractions of detected genes and reads mapping to genes, exons and in sense direction for all the three datasets (Fig. 2b). We then performed Spearman correlation analysis for pairs of datasets including control samples (Fig. 3). Correlations of RNA abundance, i.e. the number of proxy reads, between different Proximity RNA-seq datasets was found high for all pairs of datasets and controls. Similarly, we obtained good correlations between biological replicates using the number of detected co-barcoding events between two transcripts as variables. Importantly, control libraries obtained with randomly barcoded, non-clonal beads or with reverse-crosslinked RNA^12^, respectively, showed low correlation in co-barcoding to other samples. Pool 2, pool 5 and pool 8 were then merged and estimates of statistical significance for pairwise RNA proximities were derived through CloseCall Monte Carlo simulations.

To illustrate the validity of significance estimates derived by Monte Carlo simulations we first investigated the example of ribosomal RNAs (rRNA). The transcribed precursor is processed into mature 18S, 5.8S and 28S transcripts, which are then incorporated into ribosomal subunits (Fig. 4a). As expected, 18S and 28S rRNA of the small and large ribosomal subunits, respectively, showed significant RNA proximities to different precursor sequences (Fig. 4a,b). The sites of the first cleavages in pre-45S rRNA maturation are positioned in the ITS1, close to 18S, and in the 3′-ETS^19^. Interestingly, ITS1 association with 18S and 3′-ETS association with both, 18S and 28S, were not found significant, possibly due to the early cleavage and short half-life of the fulllength precursor. We note that 5.8S was poorly represented with few reads in our datasets (Fig. 4c), presumably due to low efficiency of reverse transcription in crosslinked conditions. Importantly, 5S,which is expressed from different genomic locations than pre-45S RNA, showed significant associations with 18S and 28S.

To further inspect the quality of the data we visualized RNA proximity networks of different transcripts. SNORD17 localized to nucleoli, RNase RMP with associations to nucleoli and other cellular compartments^20^, and two transcripts in the nucleoplasm more distal to nucleoli^12^. All pairwise associations (p <= 0.1) of the four transcripts were visualized as networks with edge line thickness indicating -log10 p values and node colours depicting valency, i.e. local RNA density or connectivity of transcripts (purple: high, green: low, black: middle valency grey: no valency assigned). As expected, SNORD17 was found in proximity to high valency transcripts, mostly other snoRNAs, recapitulating the RNA composition and high RNA density of nucleoli (Fig. 4d). In addition to strong associations with high valency transcripts in nucleoli, RNase MRP was also found proximal to low valency transcripts weakly or not associated to nucleoli (Fig. 4e). FGF14 and MSI2 contacted few or no high valency transcripts consistent with on average lower RNA density throughout the nucleoplasm (Fig. 4f,g).

## Usage Notes

### CloseCall dependencies

CloseCall needs the appropriate working environment and installed software tools to operate. Alternatively, the virtual machine might be used (see below). A computer with the following setup should be able to run CloseCall.

1. A Linux operating system
2. A recent version of Perl (tested on v5.10). The following non-core Perl modules should also be installed: Math::Round, String::Approx (this may require libc6-dev), PDL::LiteF, PDL::Stats (for PDL::Stats::Distr), GD::Graph (which may require libgd-gd2-perl).
3. The Perl Data Language (pdl).
4. A recent version of the sequence aligner Bowtie2 (http://bowtie-bio.sourceforge.net/bowtie2).
5. A recent version of HISAT2 (ccb.jhu.edu/software/hisat2)
6. A recent version the NGS contamination and multi-species screen tool FastQ Screen^21^
7. R (https://www.r-project.org)
8. GNU Scientific Library (GSL). We recommend using GSL 2.4.

The CloseCall master script executes the pipeline sequentially. The mapping and QC component of the pipeline may be run by typing:

perl CloseCall –map –config [configuration file] [FASTQ files]

The paths to the (i) HISAT2 reference genome index files basename, (ii) annotation gene list file and the (iii) position of the splice sites should be stored in the configuration file, as shown in the example below.

gene_list: /home/CloseCall/Data/human38_repeats_RNA45S5.txt.gz

genome: /home/CloseCall/Data/hg38_LSU_SSU_Masked_RNA45S5

splice_sites: /home/CloseCall/Data/Hg38.78.hisat2_splices.txt

On a Grid Engine compute cluster, the MonteCarlo simulation may also be run via the Perl master script, with the command:

perl CloseCall –simulations [number of simulations] [simulation formatted file]

This will qsub the jobs to the compute cluster. To generate a random dataset, specify the additional option –random.

Alternatively, the Monte Carlo simulation Jar file may be run directly with the command:

java -jar anacondamontecarlo.jar [simulation formatted file] [number of simulations]

To compare randomisations of the observed dataset against randomisations of one random dataset, i.e. the background distribution, run the command:

perl multiple_testing_correction.pl –control [random Monte Carlo results] –results [real Monte Carlo results]

To calculate the valency frequency distribution for each transcript, run the following command on the simulation formatted file of interest:

perl feature_valency_distribution.pl [simulation formatted file]

### Virtual machine and test dataset

So users may acquaint themselves with CloseCall, we have made available a virtual machine running the pipeline on a Linux operating system. All the CloseCall dependencies required for running the software have been installed on the virtual machine and there is also a test dataset, which, owing to its small size, may be processed quickly to allow users to become familiar with the various steps CloseCall undertakes when running. Having a working version of the pipeline should aid users in troubleshooting any difficulties they may have with setting up the software on their own computer. By comparing the results obtained after processing the test dataset using the virtual machine with the results generated when processing on an external device, it is possible for users to verify that their CloseCall setup is functioning correctly. The virtual machine can be obtained from the Open Science Framework website (https://osf.io/mwd73/)^13^. To that site we have also uploaded the genome index files and annotation files required for processing the test dataset, or indeed other human proximity RNA-seq samples.

The virtual machine should be run using the software VirtualBox (https://www.virtualbox.org). VirtualBox allows an operating system, with all of its installed software, to run in a special environment on top of a user’s existing operating system. VirtualBox is compatible with Windows, Linux, Macintosh, and Solaris hosts and is freely available as Open Source Software under the terms of the GNU General Public License (GPL) version 2.

## Data Availability

The code can be accessed at https://github.com/StevenWingett/CloseCall without restrictions. Specific variables or parameters used are listed in Usage Notes.
